# Esophageal Adenocarcinoma Causing Nonbacterial Thrombotic Endocarditis

**DOI:** 10.7759/cureus.39827

**Published:** 2023-06-01

**Authors:** Tosha Hedin, Mahvish Haider, Bhavtosh Dedania, Son Nguyen

**Affiliations:** 1 Internal Medicine, HCA Healthcare University of South Florida Morsani College of Medicine, Tampa, USA; 2 Gastroenterology, HCA Healthcare University of South Florida Morsani College of Medicine, Tampa, USA

**Keywords:** nonbacterial thrombotic endocarditis, tumor emboli, adenocarcinoma of esophagus (ac), barrett's esophagus (be), marantic endocarditis

## Abstract

Nonbacterial thrombotic endocarditis (NBTE) is a rare condition that causes noninfectious vegetative lesions of heart valves. NBTE is generally seen in association with advanced malignancy. The patient in this case is a 54-year-old Caucasian male with a history of rate-controlled atrial fibrillation on rivaroxaban and morbid obesity post sleeve gastrectomy in 2021, who was admitted for atrial flutter. Transesophageal echocardiogram (TEE) cardioversion was planned due to difficulty in controlling the heart rate. During the procedure, cardioversion was aborted due to TEE findings of large mobile vegetation on the left atrial side of the posterior mitral valve leaflet. The patient was afebrile for the entirety of his 10-day hospital stay, and four sets of blood cultures were negative. Further workup with esophagogastroduodenoscopy (EGD) revealed a large partially obstructing ulcerated mass in the middle and lower third of the esophagus arising in the setting of Barrett’s esophagus which was biopsy positive for esophageal adenocarcinoma. The patient was found to have advanced malignancy with metastases to the liver, adrenal glands, and perirectal lymph nodes. This case emphasizes the utilization of a TEE prior to cardioversion and also highlights the importance of EGD prior to and post gastric sleeve surgery to evaluate for esophageal cancer.

## Introduction

Nonbacterial thrombotic endocarditis (NBTE) is a rare condition that causes noninfectious lesions to previously undamaged and native heart valves. NBTE is generally associated with hypercoagulable states such as malignancy, autoimmune disease, and rarely sepsis [1]. The most common type of cancer known to cause NBTE is adenocarcinoma of the pancreas, lung, colon, ovary, and prostate [2]. These lesions can break off and cause emboli in different organs in the body, leading to cerebral vascular accidents (CVAs), myocardial infarction, or mesenteric ischemia [1]. NBTE is typically diagnosed during an autopsy [3]. If found while alive, then it is generally diagnosed during a workup to find the etiology of CVAs. We present a case of clinically asymptomatic NBTE found to be associated with metastatic esophageal adenocarcinoma.

## Case presentation

The patient is a 54-year-old Caucasian male with a history of atrial fibrillation on rivaroxaban, heart failure with preserved ejection fraction (HFpEF), and morbid obesity status post sleeve gastrectomy in 2021. The patient had no endoscopic pre or post procedure surveillance and no proton-pump inhibitor (PPI) use. He presented to the emergency department with left pleuritic chest pain radiating to the left flank after twisting his back while reaching for a high-up object. No associated diaphoresis, palpitations, nausea, abdominal pain, weight loss, or vomiting was present. On exam, his vitals showed a heart rate of 120s-140s in atrial flutter; all other vitals were stable. Physical exam was benign, including no neurological deficits. Initial labs were high-sensitivity troponin 7.0 ng/L, pro-BNP 128 pg/ml, white blood cell 6.0 x 109/L, and hemoglobin 12.0 gm/dl. 

**Figure 1:**
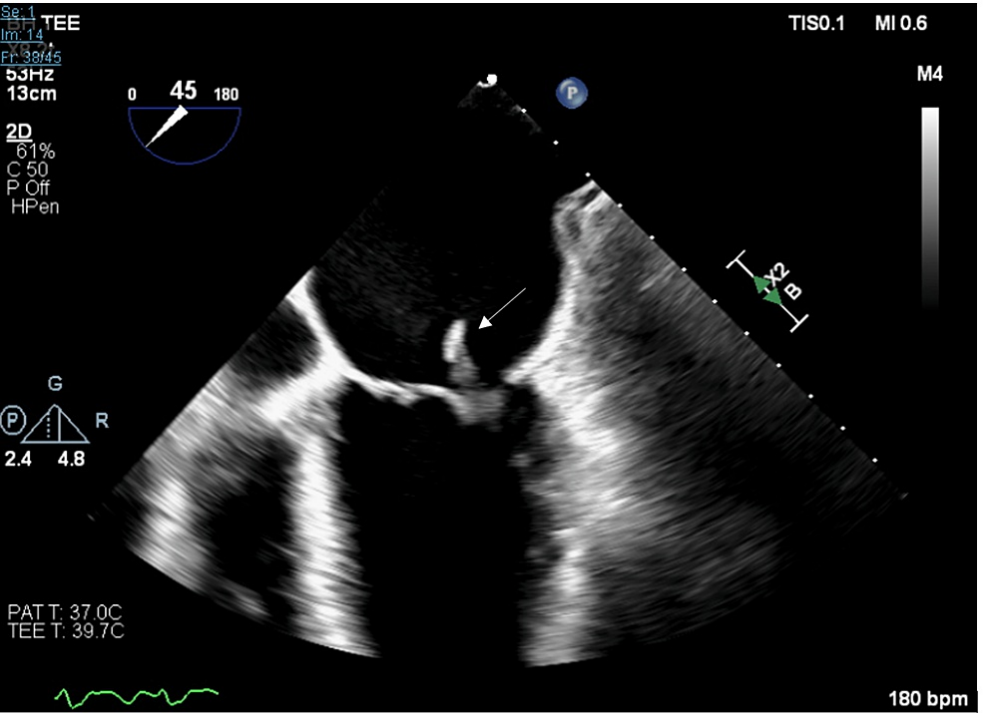
Transthoracic echocardiogram showing large mobile vegetation on the left atrial side of the posterior mitral valve leaflet.

**Figure 2:**
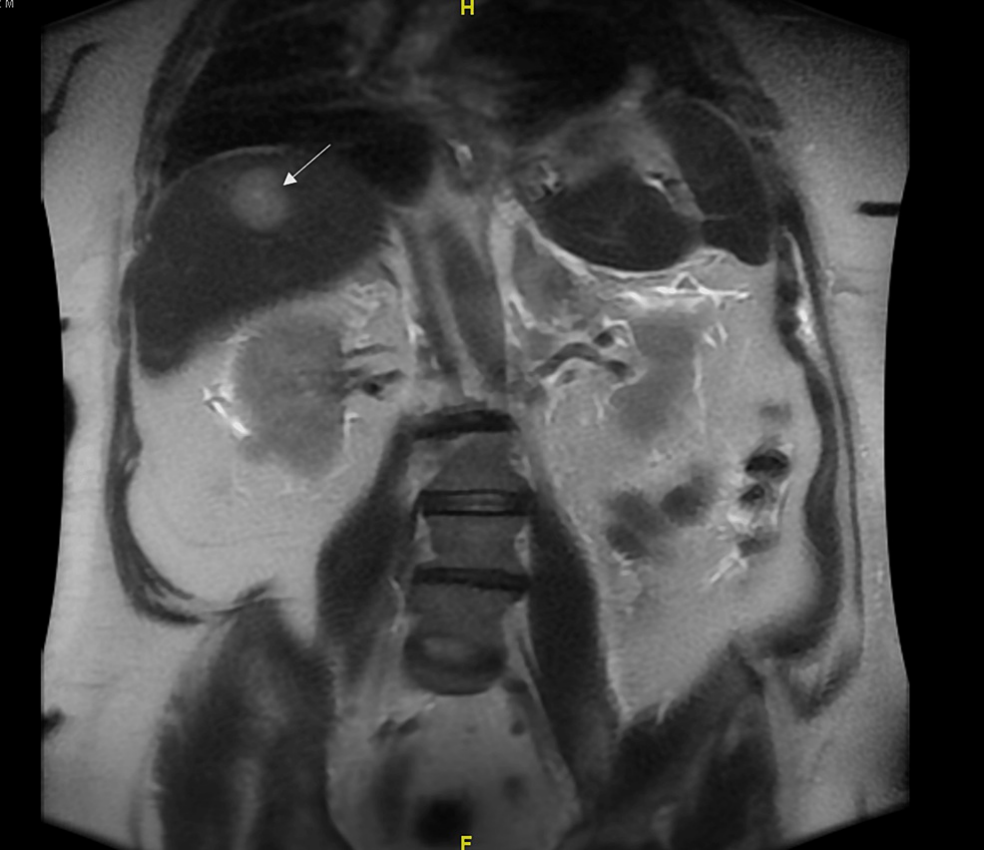
MRI of the abdomen showing a liver lesion measuring up to 3.6x3.2 cm.

Cardiology and electrophysiology physicians were consulted for a transesophageal echocardiogram (TEE) to perform cardioversion due to the persistent rapid atrial flutter despite diltiazem. TEE results showed an ejection fraction of 50% to 55%, a dilated left atrium, moderate mitral valve regurgitation with large mobile vegetation on the left atrial side of the posterior mitral valve leaflet, and a trivial pericardial effusion (Figure 1). Cardioversion was deferred due to TEE findings. CTA of the chest, abdomen, and pelvis revealed low-attenuated lesions in the liver, a 3.3 cm left adrenal nodule, and a 1.1 cm right perirectal nodule. Hematology oncology was consulted, who ordered tumor markers which showed markedly elevated Ca 19-9 2740 U/ml (reference 0-31), alpha-fetoprotein 2.6 ng/ml (reference 0-40), and carcinoembryonic antigen 11.8 ng/ml (reference 0-2.5). Subsequent MRI of the abdomen revealed several liver lesions measuring up to 3.6x3.2 cm, and multiple enlarged lymph nodes were identified in the celiac axis region, retroperitoneum, porta hepatis, and peripancreatic region. Also, a left retroperitoneal node measuring 1.2 x 2.1 cm below the level of the kidney medially and a 3.2 cm adrenal mass were observed (Figure 2). MRI brain showed two small foci of restricted diffusion in the subcortical right frontal lobe. These findings were concerning for subacute emboli. Neurology was consulted for risk stratification and the need for anticoagulation. Full-dose anticoagulation with enoxaparin was started due to the risk of further emboli being greater than the risk of hemorrhagic conversion.

**Figure 3:**
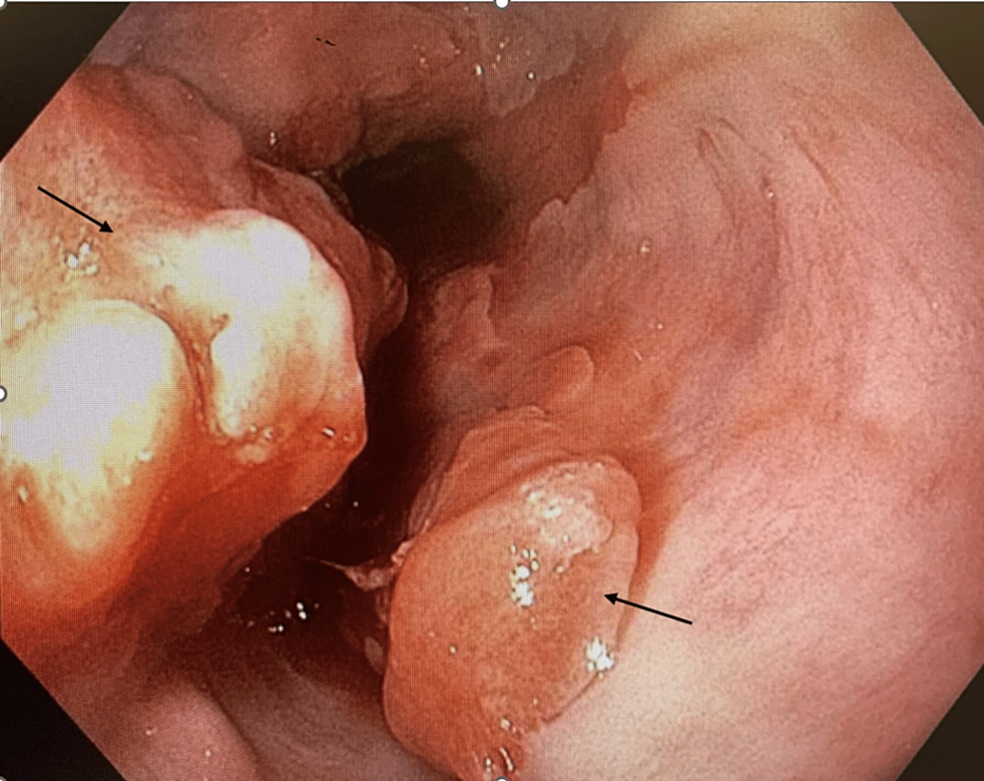
Esophagogastroduodenoscopy finding of a large ulcerating mass in the middle third and lower third of the esophagus

**Figure 4:**
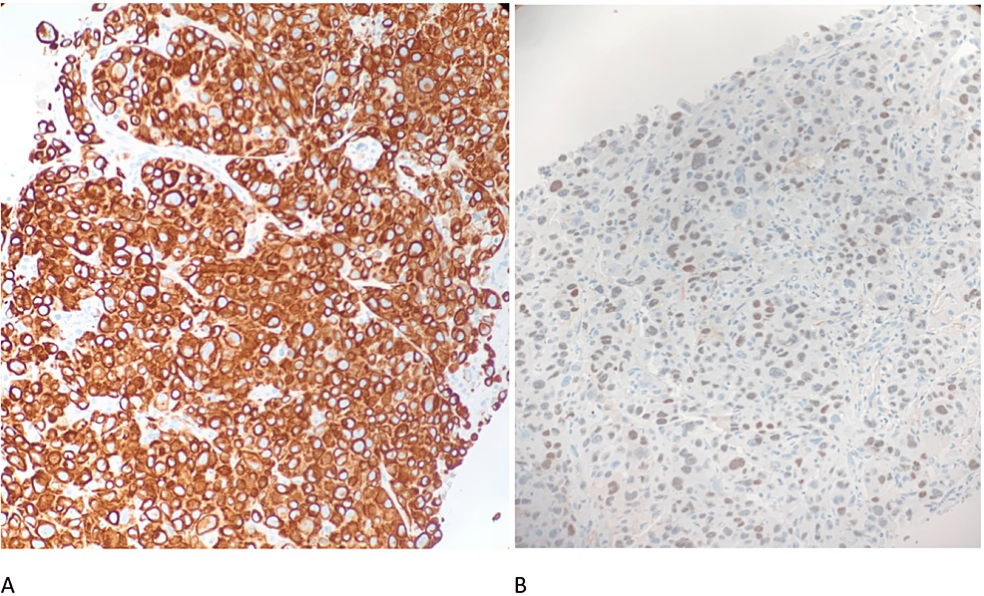
Esophageal mass biopsy showing poorly differentiated adenocarcinoma. A: CK7 immunostain positive. B: CDX-2 immunostain positive.

**Figure 5:**
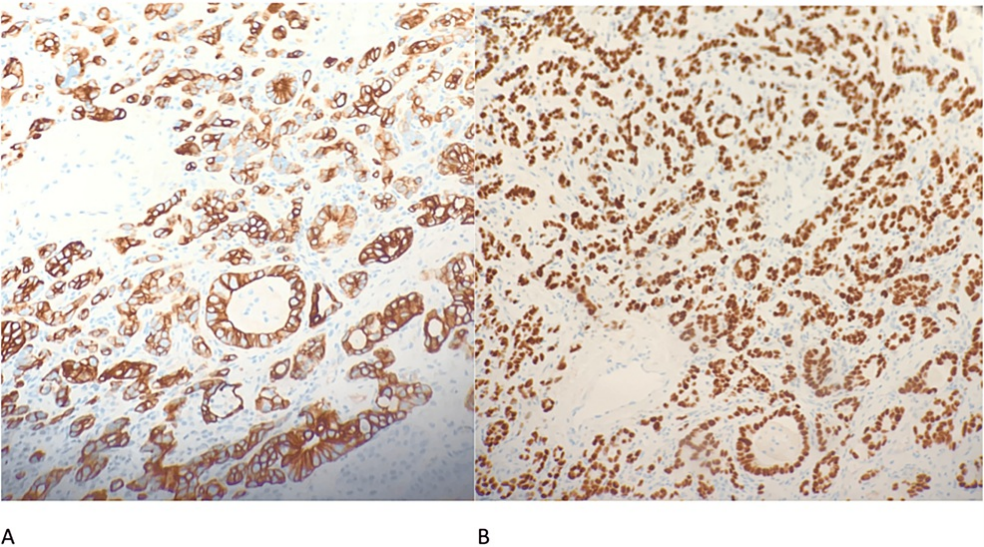
Liver mass biopsy showing poorly differentiated tumor that supports the interpretation of adenocarcinoma from upper gastrointestinal origin. A: CK7 immunostain positive. B: CDX-2 immunostain positive.

GI was consulted for an esophagogastroduodenoscopy (EGD). EGD found a large partially obstructing ulcerated mass in the middle and lower third of the esophagus arising in the setting of a large 10 cm area of suspected Barrett’s esophagus (Figure 3) and findings of sleeve gastrectomy. Pathology confirmed poorly differentiated adenocarcinoma with tumor cells staining positive for CK7 and CDX-2 immunohistochemistry (IHC) stains (Figure 4). Liver biopsy revealed similar morphology (Figure 5) and the IHC staining pattern with medium to large cells with amphophilic cytoplasm and cytologic high-grade nuclei with scattered large, bizarre forms. These findings suggest adenocarcinoma originating from the upper gastrointestinal tract and less likely pancreatic biliary origin. Rheumatologic workup was incidentally positive for weak antinuclear antibody and scl-70 scleroderma antibody. During the entire 10-day hospital course, the patient remained afebrile, and four sets of blood cultures were negative for any growth of bacteria. Even though there was a low index of suspicion, the patient was started on empiric ceftriaxone on hospital day eight, with no clinical improvement. He was discharged with a six-week course of ceftriaxone and recommended follow-up with an oncologist for palliative chemotherapy. His atrial flutter was rate controlled with oral metoprolol succinate and digoxin. The patient’s condition continued to decline while at home. He elected to go to hospice a week after discharge and passed away five days later.

 

## Discussion

NBTE is a rare condition that consists of noninfectious vegetative lesions of the heart valve. The vegetation is made from thrombi intermixed with strands of fibrin, platelets, mononuclear cells, and immune complexes. The vegetation is without bacteria or inflammation [4]. This mix of substances causes the vegetation to be friable and easily embolize, like the patient in our case. Our patient’s presentation was rare due to the absence of any neurological deficits from the subacute brain emboli as seen on MRI.

NBTE is generally seen in association with advanced malignancy, which comprises 80% of cases [5]. In a small study including 10 cases of NBTE, eight patients had a malignant tumor. The most common type of malignancy was adenocarcinoma [5]. Of the adenocarcinoma subgroup, lung, pancreas, stomach, and adenocarcinomas of unknown primary sites were the most common in descending order [6]. Our patient has a unique diagnosis because he had esophageal adenocarcinoma.

Esophageal adenocarcinoma is the most common subtype among all esophageal cancer types in the United States. The most important risk factor is the presence of long-standing Barrett’s esophagus, which causes a 30-fold increased risk compared to the average population [7]. Risk factors for Barrett’s esophagus are chronic gastroesophageal reflux disease (GERD), obesity, smoking, Caucasian race, and male sex. Our patient met all the major risk factors, except for smoking tobacco. He was particularly vulnerable to Barrett’s esophagus due to his gastric sleeve surgery. Untreated GERD post gastric sleeve increases the development incidence of Barrett’s esophagus and exacerbates symptoms of GERD [8]. In a systemic review, 17 esophagogastric cancers after gastric sleeve were identified. Tumors were diagnosed after an interval of 33.9 ±22.8 months from gastric sleeve. Adenocarcinoma was the most frequent tumor histology at 88.2% [8]. This patient should have had an EGD prior to gastric sleeve surgery to determine if he was a good fit for the procedure, considering he may have had Barrett’s esophagus even prior to his surgery. He never complained of GERD symptoms. On review of his home medication list, the patient was not on any PPI or histamine type-2 receptor antagonist for chronic acid suppression therapy.

Most cases of esophageal adenocarcinoma are diagnosed in late stages due to the lack of symptoms. If a patient does have symptoms, it may be dysphagia, odynophagia, weight loss, or rarely heartburn [7]. These vague warning signs are likewise side effects of gastric sleeve surgery. NBTE also has late presenting symptoms. In fact, most patients are diagnosed during an autopsy. If NBTE is found during life, the most common presentation is a focal neurological deficit due to emboli in the brain [3].

Definitive treatment includes removing the thrombus, but these patients are typically not surgical candidates due to overall poor prognosis from metastatic cancer [3]. Treatment with heparin-based anticoagulation and chemotherapy has been shown to reduce risk of thromboembolic episodes. Our patient was treated with a full dose of enoxaparin.

A high index of suspicion is necessary for accurate diagnosis. In this case, there was an absence of fever and heart murmur, four sets of negative blood cultures, and a lack of improvement after antibiotic therapy. The only way to definitively diagnose NBTE is histology through specimen acquisition [4]. This is rarely done while the patient is alive. Also, by the time patients present for medical care, it is late in their disease process. Our patient had no deficits but did have multiple metastases which were incurable and ultimately fatal. 

## Conclusions

This case stresses the importance of multiple clinical practice changes physicians can adopt. Firstly, TEE prior to planned cardioversion can prevent emboli in the brain. This thrombus would have likely caused a catastrophic CVA if cardioversion would have been performed without due diligence. Secondly, routine pre and post EGD after a bariatric surgery can be done to screen for Barrett’s esophagus. Third, routine screening of Barrett’s esophagus in patients at risk for esophageal carcinoma can be conducted to identify early cancers. Finally, the importance of chronic acid suppression in people with chronic GERD or risk factors for Barrett’s esophagus is highlighted. 
